# Complete Genome Sequence of *Halomonas* sp. Strain SH5A2, a Dye-Degrading Halotolerant Bacterium Isolated from the Salinas and Aguada Blanca National Reserve in Peru

**DOI:** 10.1128/MRA.01083-20

**Published:** 2021-01-14

**Authors:** Walter F. Manya, Wendy C. Lizárraga, Carlo G. Mormontoy, Mario A. Taira, Pablo S. Ramírez

**Affiliations:** a Laboratory of Molecular Microbiology and Biotechnology, Faculty of Biological Sciences, Universidad Nacional Mayor de San Marcos, Lima, Peru; Indiana University, Bloomington

## Abstract

*Halomonas* sp. strain SH5A2 is a halotolerant bacterium isolated from Salinas Lake at 4,300 m above sea level in Peru. Here, we report its complete genome sequence with a length of 3,849,224 bp and highlight the presence of genes putatively related to dye degradation, such as NADPH-dependent oxidoreductases.

## ANNOUNCEMENT

*Halomonas* strains are Gram-negative, rod-shaped, aerobic, and halotolerant bacteria (1). Many strains grow and decolorize harmful synthetic azo dyes in anaerobic cultures with up to 20% (wt/vol) NaCl (2–4). Moreover, enzymes with azoreductase function were characterized in this genus (5, 6).

A novel strain of *Halomonas* (SH5A2) was isolated in plates with seawater medium (pH 7.5) with 12.5% NaCl from a 10% precultured sample in seawater broth (7) collected from Salinas Lake (16°20′10″S, 71°08′30″W) within the Salinas and Aguada Blanca National Reserve in Arequipa (Peru) and incubated at 30°C under static conditions for 48 h.

For DNA extraction, strain SH5A2 was cultivated in seawater broth at 30°C under static conditions for 48 h to the mid-log phase (8). This culture was used for DNA extraction with the Wizard genomic DNA purification kit (Promega, USA) following the manufacturer’s instructions. Library construction prepared inserts of 20 kb for single-molecule real-time (SMRT) sequencing on the Pacific Bioscience RS II platform. Raw sequencing data included 116,274 subreads encompassing 1,272,266,623 bp. The average subread length and *N*_50_ value were 10,941 bp and 16,510 bp, respectively. The Canu v2.0 assembler (9) corrected and trimmed reads to produce 5,379 reads with 129,094,014 bp, which were used to perform *de novo* assembly into a single contig (correctedErrorRate=0.039). Subsequently, the Arrow algorithm in the GenomicConsensus v2.3.3 package (Pacific Biosciences, USA) was chosen to improve genomic sequence quality through several polishing rounds. The genome circularization was performed with Circlator v1.5.5 using corrected reads and the polished assembly (10) and visualized with the CGView Server (11).

Annotation was performed with the RAST v2.0 server (12), Prokka v1.14.0 (13), and NCBI Prokaryotic Genome Annotation Pipeline (PGAP) v4.12 (14). In addition, to perform a phylogenetic analysis, a nucleotide sequence alignment was made with MAFFT v7.471 (https://mafft.cbrc.jp/alignment/software), followed by the best nucleotide substitution model prediction (HKY+F+R2) and phylogenetic tree construction using the 16S rRNA gene with IQ-TREE v1.6.12 software (15). Finally, tree visualization was performed with FigTree v1.4.4 (http://tree.bio.ed.ac.uk/software/figtree). Default parameters were used except where otherwise noted.

**FIG 1:**
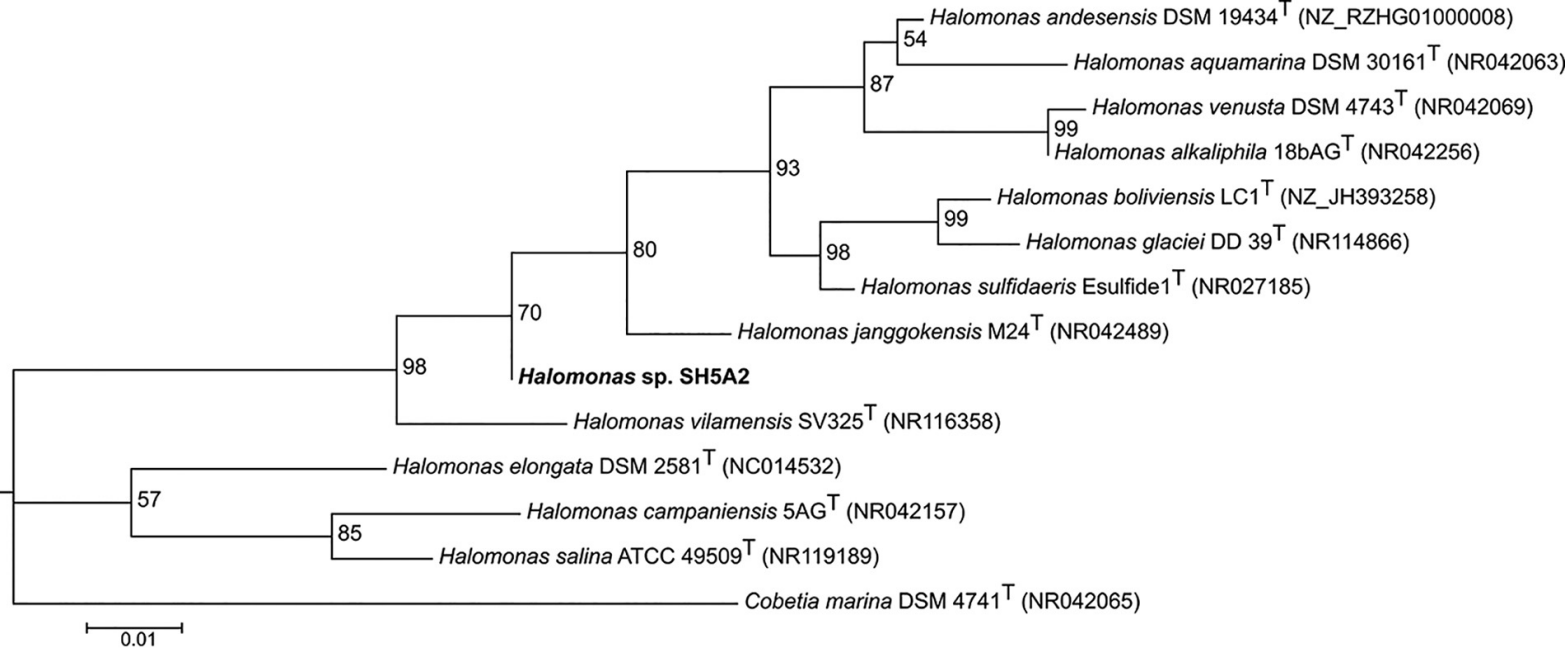
Phylogenetic tree of the 16S rRNA gene in *Halomonas* sp. SH5A2 based on the maximum likelihood method with 1,000 bootstraps. GenBank accession numbers are listed in parentheses.

Summary statistics and characteristic features of the complete genome sequence of *Halomonas* sp. strain SH5A2 are given in Table 1. This genome mainly encodes proteins related to carbohydrate metabolism, and it has 46 proteins related to xenobiotic metabolism and biodegradation. Genes encoding proteins putatively related to dye degradation were predicted as NADPH-dependent oxidoreductases (HXW73_11090 and HXW73_16825) and a pseudogene for NAD(P)H-dependent oxidoreductase protein (HXW73_07605) previously identified by RAST as a flavin mononucleotide (FMN)-dependent NADH-azoreductase. The phylogenetic tree shown in Fig. 1 allows us to determine that strain SH5A2 diverges significantly from other species, forming a separate group that could belong to a new species. We conclude that *Halomonas* sp. SH5A2 could have great potential in bioremediation of textile effluents due to its capacity to tolerate high NaCl concentrations and the presence of genes putatively involved in dye degradation.

**TABLE 1:** Summary statistics and characteristic features of strain SH5A2 genome sequencing

Characteristic	Data for *Halomonas* sp. SH5A2
BioProject accession no.	PRJNA639301
BioSample accession no.	SAMN15395493
No. of subreads	116,274
Subread *N*_50_ (bp)	16,510
Genome size (bp)	3,849,224
G+C content (%)	56.9
Coverage (×)	33.44
Total no. of genes	3,578
No. of coding sequences	3,495
No. of rRNAs	18
No. of tRNAs	64

### Data availability.

The genome sequence and raw data reported here were deposited in the GenBank (accession number CP058321) and SRA (accession number SRR12147485) databases, respectively.
